# Evaluating diagnostic accuracy and agreement of TI-RADS scoring in thyroid nodules: A comparative analysis between sonographers and radiologists

**DOI:** 10.1371/journal.pone.0312121

**Published:** 2024-10-11

**Authors:** Abdulrahman M. Alfuraih, Abdullah M. Alotaibi, Alanoud K. Alshammari, Basmah F. Alrashied, Yahya M. Mashhor, Mustafa Mahmoud, Mohammed J. Alsaadi

**Affiliations:** 1 Radiology and Medical Imaging Department, College of Applied Medical Sciences, Prince Sattam bin Abdulaziz University, Kharj, Saudi Arabia; 2 Research Center, King Fahad Medical City, Riyadh, Saudi Arabia; 3 Ultrasound Unit, Radiology Department, King Fahad Medical City, Riyadh, Saudi Arabia; 4 Radiology Department, Altakassusi Alliance Medical, Riyadh, Saudi Arabia; 5 Department of Radiological Sciences, College of Applied Medical Sciences, King Khalid University, Abha, Saudi Arabia; 6 Radiology and Medical Imaging Department, College of Applied Medical Sciences, Prince Sattam bin Abdulaziz University, Kharj, Saudi Arabia; Neyshabur University of Medical Sciences, ISLAMIC REPUBLIC OF IRAN

## Abstract

**Objective:**

The Thyroid Imaging Reporting and Data System (TI-RADS) is an essential tool for assessing thyroid nodules, primarily used by radiologists. This study aimed to compare the agreement of TI-RADS scores between sonographers and radiologists and to assess the diagnostic performance of these scores against histological findings in suspicious thyroid nodules.

**Methods:**

In a retrospective analysis, 168 patients with suspicious thyroid nodules classified as TR3 and above by the radiologists were included. Both sonographers and radiologists independently assigned the American College of Radiologists (ACR) TI-RADS scores, which were then compared for inter-reader agreement using Cohen’s Kappa statistic. The scores were also evaluated for diagnostic performance against histological results based on the Bethesda system.

**Results:**

The study revealed a moderate overall agreement between sonographers and radiologists in TI-RADS scoring (κ = 0.504; 95% CI: 0.409–0.599), with poor agreement noted specifically for nodule margin scores (κ = 0.102; 95% CI: -1.430–0.301). In terms of diagnostic performance against histological outcomes, sonographers’ TI-RADS scores showed a sensitivity of 100% and a specificity of 44.6%, while radiologists’ scores showed a sensitivity of 100% but a lower specificity of 29.3%.

**Conclusion:**

The findings indicate moderate agreement in TI-RADS scoring between sonographers and radiologists, with reproducibility challenges especially in scoring nodule margins. The marginally superior diagnostic performance of sonographers’ scores suggests potential efficiency benefits in involving sonographers in preliminary assessments. Future research should aim to encompass a wider range of TI-RADS categories and focus on minimizing scoring variability to enhance the system’s clinical utility.

## Introduction

Thyroid nodules are a common clinical finding, with a prevalence of 19–68% in the general population when assessed by ultrasound [1]. The majority of these nodules are benign, and only a small percentage are malignant [2]. Accurate identification of high-risk nodules is crucial for timely intervention and optimal patient management. The Thyroid Imaging Reporting and Data System (TI-RADS) is a widely used classification system for the risk stratification of thyroid nodules based on ultrasound features [3,4]. This system aims to standardize the reporting of thyroid ultrasound findings and improve the diagnostic accuracy of thyroid nodule evaluation [5,6].

The rationale behind this study is rooted in the clinical need for accurate scoring of thyroid nodules to guide patient management. High-risk nodules may require fine needle aspiration (FNA) to rule out malignancy, whereas low-risk nodules can be managed conservatively without invasive procedures [7,8]. Accurate TI-RADS scoring is essential for identifying which patients require FNA and sparing those with low-risk nodules from unnecessary invasive procedures.

Multiple previous studies have demonstrated a high accuracy of sonographers’ diagnostic opinion compared to radiologists in abdominal [9–16], obstetric [17], musculoskeletal [18], and emergency ultrasound [19]. However, no previous studies have directly compared the agreement of TI-RADS scores between sonographers and radiologists. Albeit previous studies have examined the agreement of TI-RADS scores between radiologists, with varying results [20–25]. Given the increasing demand for ultrasound examinations and the limited availability of radiologists, it is essential to determine if sonographers can accurately assess thyroid nodules using the TI-RADS scoring system [26,27]. Moreover, if sonographers can reliably and accurately score thyroid nodules, radiologists may be able to focus on more complicated tasks, improving overall efficiency and resource allocation within the radiology department [28,29].

Despite the potential benefits of using TI-RADS in clinical practice, some concerns have been raised regarding the reproducibility and reliability of the classification system [30]. Inconsistencies in the application of TI-RADS criteria and interobserver variability may affect the accuracy of the scoring system, warranting further investigation to ensure its validity and reliability [31].

The main objectives of this study are twofold. The first is to compare agreement of TI-RADS scores assigned by sonographers and radiologists. The second objective is to analyze the diagnostic accuracy of the TI-RADS scoring system. The underlying hypothesis is that the scores assigned by sonographers agree with those assigned by radiologists. If confirmed, this could have considerable implications for the allocation of resources within radiology.

## Materials and methods

### Study design and case selection

The study employed a retrospective design, covering the period from January 2021 to January 2022. The research data were accessed from the radiology department at King Fahad Medical City in Riyadh, Saudi Arabia, between July 2022 and January 2023 for research purposes. To minimize selection bias, we included all consecutive adult patients who met the inclusion criteria during the study period, ensuring a representative sample. The study was approved by the hospital’s IRB committee and a consent waiver was granted (NIH registration number: IRB00010471) to retrospectively collect this study data.

During the study period, all patients with thyroid nodules received a TI-RADS score [according to the TI-RADS system developed by the American College of Radiologists (ACR) [8]] by the scanning sonographer and the reporting radiologist in real-time during the patient visits, which was documented on the radiology information system (RIS). Each case was scored based on two-plane grey-scale images of the thyroid nodule. When a patient has more than one nodule, the most suspicious one was scored and documented. The patients were considered eligible if they were an adult (18 years or older) and had a documented Bethesda score following an FNA biopsy done after the ultrasound scan. Patients who did not undergo thyroid sonography prior to the FNA or the duration between the scan and FNA was more than two weeks were excluded.

### TI-RADS scoring

The thyroid ultrasound scans were performed using a linear array transducer (5–15 MHz) on a LOGIQ E9 ultrasound system (GE Healthcare, Milwaukee, WI, USA) by one of 11 accredited sonographers with ultrasound experiences ranging from 2 to 18 years. All sonographers were trained to interpret thyroid US images and assign ACR TI-RADS scores after receiving a focused training course by the chief consultant radiologist (12 years of experience). The training was done in July 2020, which included official educational materials developed by the ACR including the TI-RADS atlas and user guide [32]. This was followed by regular monitoring and feedback over 3 months. The retrospective data collected for this study was set after approximately 6-months of the sonographers’ training and practice. Hence, all sonographers had the same level of experience (6-months) in TI-RADS scoring. Following the sonographers TI-RADS scores, the images were reviewed by one of 5 accredited radiologists with experiences ranging from 5 to 12 years and a TI-RADS score was documented on the RIS system. The radiologists included in the study consisted of three neuroradiologists, one cardiothoracic radiologist, and one abdominal radiologist. While their primary subspecialties are varied, all radiologists are board-certified and routinely interpret thyroid ultrasound examinations as part of their clinical duties.

### Bethesda scoring

After the ultrasound examinations, fine-needle aspiration (FNA) biopsies were performed on suspicious thyroid nodules (≥ TR3 score by the radiologist). The cytological results were reported according to the Bethesda System for Reporting Thyroid Cytopathology (TBSRTC) [33]. This standardized system categorizes thyroid FNA results into six diagnostic categories: Category I: Non-diagnostic or Unsatisfactory; Category II: Benign; Category III: Atypia of Undetermined Significance or Follicular Lesion of Undetermined Significance; Category IV: Follicular Neoplasm or Suspicious for a Follicular Neoplasm; Category V: Suspicious for Malignancy; Category VI: Malignant.

FNA procedures were conducted by experienced interventional radiologists under ultrasound guidance using a 25-gauge needle. The obtained samples were processed and evaluated by board-certified cytopathologists following the TBSRTC guidelines. For our analysis, we grouped the Bethesda categories as follows: Benign: Category II; Malignant or Suspicious for Malignancy: Categories V and VI; Indeterminate: Categories I, III, and IV [25,33].

### Statistical analysis

All categorical variables are presented as frequencies and percentages while continuous variables are presented as mean ± SD. The Kolmogorov-Smirnov test was used to confirm the assumption of normal distribution. The Bethesda scores were used to categorize the nodules into benign (Bethesda II), suspicious or malignant (Bethesda V and VI), or indeterminate (Bethesda I, III, and IV) [25,33].

Inter-reader reliability for TI-RADS scores were assessed using Cohen’s Kappa statistic. The Kappa scale was interpreted as follows: <0, poor agreement; 0.01–0.20, slight agreement; 0.21–0.40, fair agreement; 0.41–0.60, moderate agreement; 0.61–0.80, substantial agreement; and 0.81–1.00; almost perfect agreement. Diagnostic accuracy analysis was performed to determine the sensitivity and specificity analysis of TI-RADS by sonographer and radiologist. To simplify the analysis, TR1 and TR2 were categorized as “low suspicion of malignancy” and TR3, TR4, TR5 categorized as “high suspicion of malignancy” [34]. The indeterminate (Bethesda I, III, and IV) scores were excluded from the diagnostic accuracy analysis. Receiver Operating Characteristic (ROC) curve analyses were conducted to evaluate and compare the diagnostic performance of the sonographers and radiologists. The area under the curve (AUC) was calculated for each group to quantify their overall diagnostic accuracy. The association between TI-RADS scores and the histological outcomes was evaluated using the Chi-Square test. A two-sided p-value less than 0.05 was considered statistically significant. All data was entered and analyzed using the SPSS 25 Statistics Package (SPSS Inc., Chicago, Illinois, USA) and MEDCALC version 18.11.6 (Acacialaan 22 8400 Ostend Belgium).

## Results

**Fig 1:**
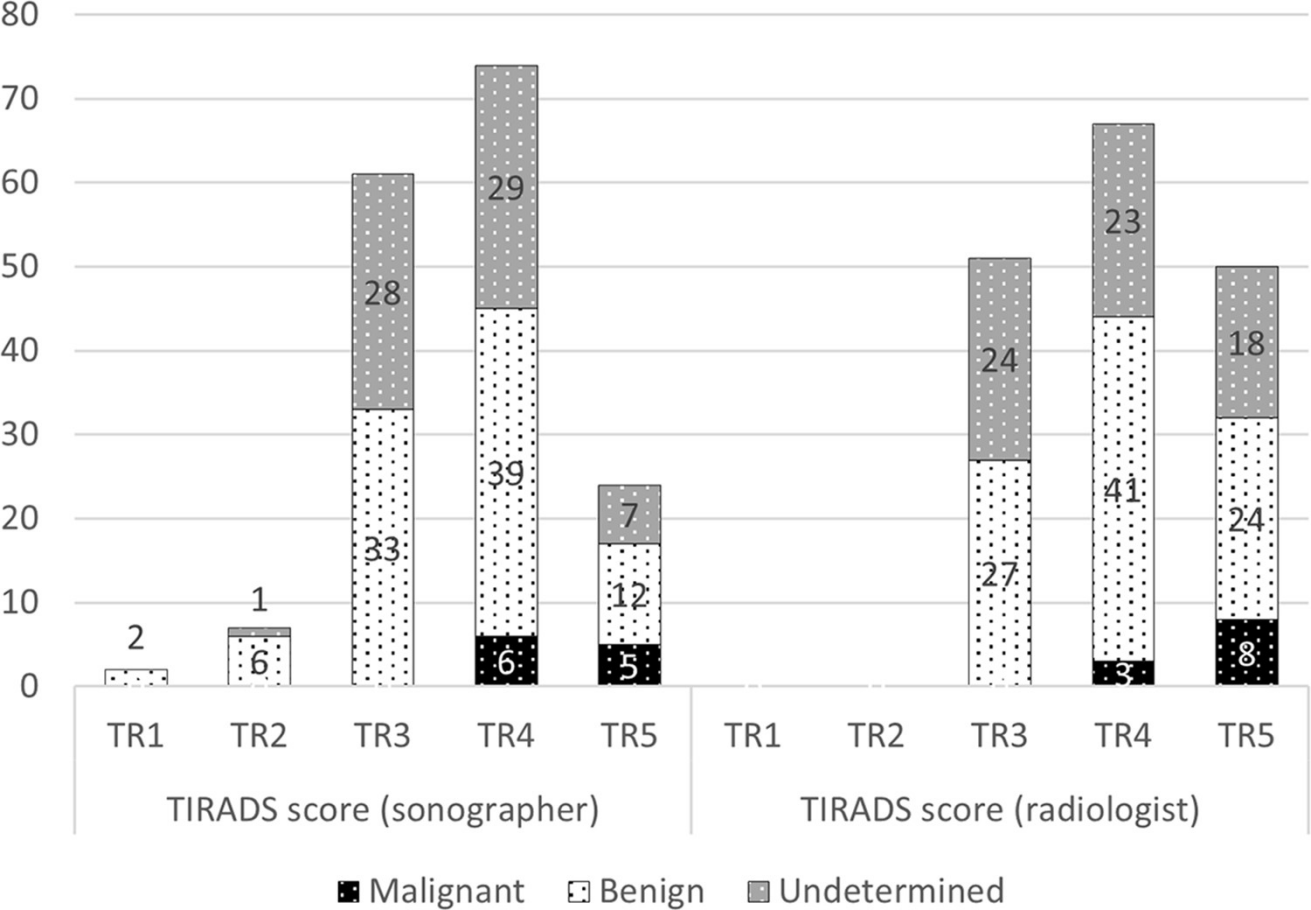
Overall distribution of the TI-RADS scores of the sonographer and radiologist.

A total of 168 patients were eligible (adults with available TI-RADS and Bethesda scores) and included in the study, of which 18 (11.1%) were male and 150 (88.9%) were female. The mean age was 50.3 ± 13.7 years. The histological results of the nodules were as follows: malignant or suspicious nodule (n = 11), benign nodule (n = 92), and indeterminate nodule (n = 65). The distribution of TI-RADS scores and Bethesda scores is presented in Table 1 and Fig 1 respectively. The 9 patients scored by the sonographers as TR1 or TR2 had benign follicular nodules (Bethesda II). The detailed distribution of the TI-RADS features based on the Bethesda subcategories is available in the supplementary materials (S1 Table). All 11 patients with FNA results indicating malignancy (n = 8) or suspicion for malignancy (n = 3) underwent surgical excision and histopathological examination. The histopathological analysis confirmed malignancy in all cases, showing concordance with the FNA cytology results.

**Table 1:** Distribution of thyroid nodules based on the Bethesda results and TI-RADS scores.

	Description	Nodules		
		Malignant or Suspicious Nodule (n = 11)	Benign Nodule (n = 92)	Indeterminate Nodule (n = 65)
FNA—Bethesda score	I. Non-diagnostic or Unsatisfactory	0	0	16 (24.6%)
	II. Benign	0	92 (100.0%)	0
	III. Atypia of Indeterminate Significance or Follicular Lesion of Indeterminate Significance	0	0	43 (66.2%)
	IV. Follicular Neoplasm or Suspicious for a Follicular Neoplasm	0	0	6 (9.2%)
	V. Suspicious for Malignancy	3 (27.3%)	0	0
	VI. Malignant	8 (72.7%)	0	0
TI-RADS score (sonographer)	TR1	0	2 (2.2%)	0
	TR2	0	6 (6.5%)	1 (1.5%)
	TR3	0	33 (35.9%)	28 (43.1%)
	TR4	6 (54.5%)	39 (42.4%)	29 (44.6%)
	TR5	5 (45.5%)	12 (13.0%)	7 (10.8%)
TI-RADS score (radiologist)	TR1	0	0	0
	TR2	0	0	0
	TR3	0	27 (29.3%)	24 (36.9%)
	TR4	3 (27.3%)	41 (44.6%)	23 (35.4%)
	TR5	8 (72.7%)	24 (26.1%)	18 (27.7%)

* Categorical data presented as frequency (%).

### Agreement results

**Fig 2:**
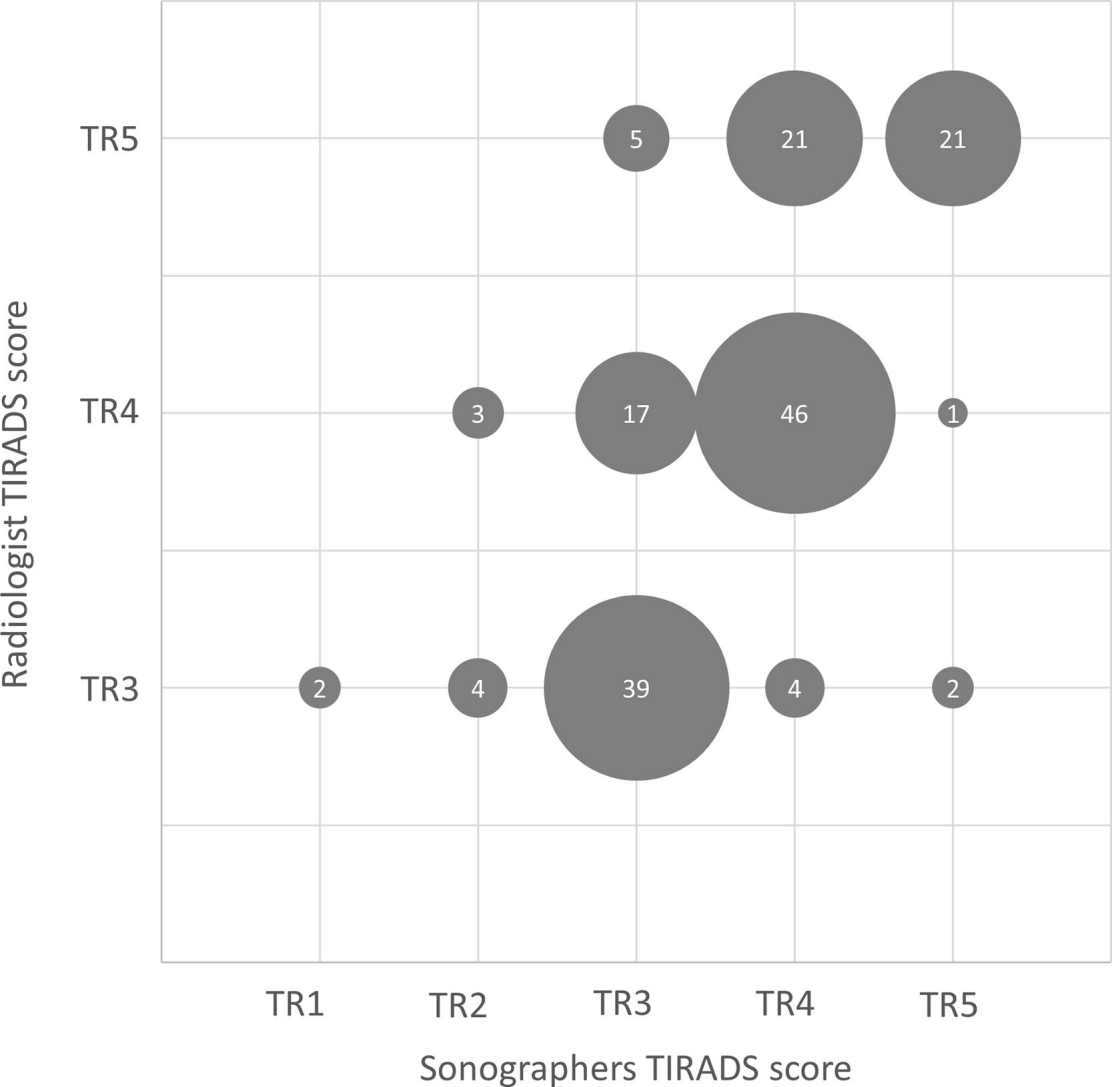
Bubble chart of readers’ agreement on TI-RADS scores. The Bubble labels represent the instances of matched scores.

Table 2 cross-tabulates the scores by the radiologists and sonographers’ readers per case. The scores are also visually presented in Fig 2. The results demonstrate good agreement overall, and few instances of substantial discrepancies in scoring between readers. For example, two cases where scored TR1 by the sonographer compared to TR3 by the radiologist.

**Table 2:** Cross-tabulation of readers’ agreement on TI-RADS scores shown as count and frequency.

		Sonographers TI-RADS					
		TR1	TR2	TR3	TR4	TR5	Total
Radiologists TI-RADS	TR3	2 (1.2%)	4 (2.4%)	39 (23.2%)	4 (2.4%)	2 (1.2%)	51 (30%)
	TR4	-	3 (1.8%)	17 (10.1%)	46 (27.4%)	1 (0.6%)	67 (40%)
	TR5	-	-	5 (3.0%)	24 (14.3%)	21 (12.5%)	50 (30%)
	Total	2 (1.2%)	7 (4%)	61 (36%)	74 (44%)	24 (14%)	168 (100%)

Inter-reader reliability was assessed using the Kappa statistic in Table 3. Moderate agreement was found between the sonographer and radiologist readers for TI-RADS (κ = 0.504), composition score (κ = 0.463), echogenicity score (κ = 0.584), shape score (Kappa = 0.462), and echogenic foci score (κ = 0.548) (Table 3). All p-values were significant at p < 0.001, indicating significant agreement among readers. The margin score, however, was not significant (p = 0.153) and the Kappa statistic of 0.102 indicated slight agreement.

**Table 3:** Inter-reader reliability between sonographer and radiologist readers of Thyroid Imaging Reporting and Data System (TI-RADS) features.

Feature	Kappa	SE	95% C.I	P–value
TI-RADS	0.504	0.049	[0.409–0.599]	*<0.001
Composition score	0.463	0.091	[0.283–0.642]	*<0.001
Echogenicity score	0.584	0.054	[0.478–0.689]	*<0.001
Shape score	0.462	0.131	[0.204–0.719]	*<0.001
Margin score	0.102	0.102	[-1.430–0.301]	0.153
Echogenic foci score	0.548	0.052	[0.446–0.650]	*<0.001

* shows that P-value is significant at P<0.05 and agreement among participants, CI, confident Interval; SE, standard error.

### Diagnostic accuracy results

In evaluating the diagnostic accuracy of TI-RADS scores in Tables 4 and 5, in which the indeterminate nodules were excluded, both sonographers and radiologists demonstrated high sensitivity but varied in specificity and positive likelihood ratios. The sonographers achieved a sensitivity of 100% (95% CI: 71.5–100.0), indicating accurate identification of all positive (suspicious or malignant) cases (11 out of 11). However, specificity was moderate at 44.6% (95% CI: 34.2–53.3), suggesting a higher rate of false-positive diagnoses compared to true negatives. The positive likelihood ratio was 1.80 (95% CI: 1.5–2.2), reinforcing the reliability of a positive test result in diagnosing the disease. The positive predictive value (PPV) was 17.7% (95% CI: 15.2–20.6), and the overall accuracy of the sonographer’s TI-RADS scoring was 50.5% (95% CI: 40.5–60.5).

**Table 4:** Contingency tables for the diagnostic accuracy of Thyroid Imaging Reporting and Data System (TI-RADS) scores by sonographer and radiologist.

		Nodule histology diagnosis		P—value
		Malignant (n = 11)	Benign (n = 92)	
**TI-RADS (Sonographers)**	Suspicious scores (TR4, TR5)	11 (100%)	51 (55.4%)	*0.003
	Negative or low suspicion scores (TR1, TR2, TR3)	0 (0%)	41 (44.6%)	
**TI-RADS (Radiologists)**	Suspicious scores (TR4, TR5)	11 (100%)	65 (70.7%)	*0.029
	Negative or low suspicion scores (TR1, TR2, TR3)	0 (0%)	27 (29.3%)	

Note: * shows that P-value is significant at P<0.05.

**Table 5:** Diagnostic performance of Thyroid Imaging Reporting and Data System (TI-RADS) scores by sonographer and radiologist.

	Sonographers		Radiologist	
Statistic	Value	95% CI	Value	95% CI
**Sensitivity**	100.0%	[71.5–100.0]	100.0%	[71.5–100.0]
**Specificity**	44.6%	[34.2–53.3]	29.3%	[20.3–39.8]
**Positive Likelihood Ratio**	1.8	[1.5–2.2]	1.4	[1.2–1.6]
**Positive Predictive Value**	17.7%	[15.2–20.6]	14.5%	[12.9–16.2]
**Negative Predictive Value**	100.0%	-	100.0%	-
**Accuracy**	50.5%	[40.5–60.5]	36.90%	[27.6–47.0]

Similarly, the radiologist’s sensitivity was 100% (95% CI: 71.5–100.0). However, specificity was lower at 29.3% (95% CI: 20.3–39.8), indicating a greater tendency for false positives with only 27 of 92 non-malignant cases correctly identified. The positive likelihood ratio of 1.4 (95% CI: 1.2–1.6) was also lower compared to the sonographer, suggesting a slightly reduced diagnostic accuracy in positive test interpretation. The PPV was 14.5% (95% CI: 12.9–16.2), and the overall accuracy was 36.9% (95% CI: 27.6–47.0). The negative predictive value (NPV) was 100% for both sonographers and radiologists, indicating that all nodules with negative or low suspicion TI-RADS scores were confirmed to be benign on histology when tested.

**Fig 3:**
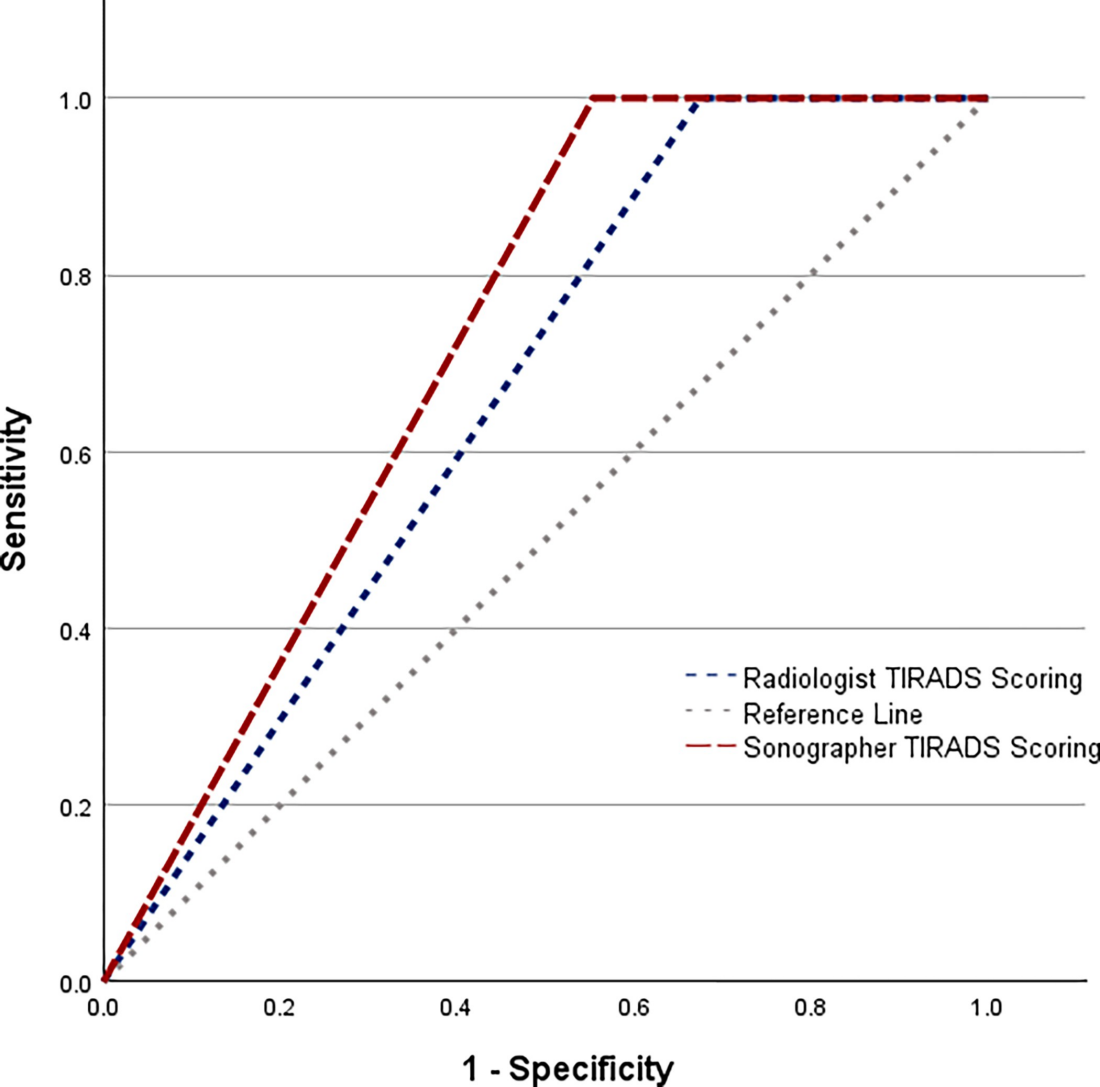
ROC curves comparing the diagnostic performance of sonographers and radiologists.

The ROC curves for sonographers and radiologists are presented in Fig 3. The AUC for sonographers was 0.723 (95% CI: 0.684–0.762), indicating acceptable diagnostic accuracy. For radiologists, the AUC was 0.662 (95% CI: 0.626–0.699). The difference between the two AUCs was statistically significant (p < 0.001), suggesting that sonographers had a slightly better diagnostic performance compared to radiologists.

## Discussion

This study is the first, to our knowledge, that compared the agreement of TI-RADS scores in suspicious thyroid nodules between sonographers and radiologists. Our results highlighted an overall moderate agreement between the two groups of readers. This agrees with the published results on inter-reader reproducibility between radiologists in other studies. For example, Liu et al. [23] conducted a meta-analysis of seven studies investigating the agreement of TI-RADS scores between various groups of radiologists. They found an overall moderate agreement across the seven studies (κ = 0.54; 95% CI: 0.49−0.58). More recently, Li et al [35] conducted a similar meta-analysis, which yielded a total of 13 studies. The pooled inter-reader agreement for overall ACR TI-RADS system was also moderate (k = 0.51, 95% CI: 0.42–0.59). They reported no significant difference between experienced and inexperienced readers (p = 0.55). They also noted that agreements were usually higher in inexperienced readers, which could be explained by their likelihood to follow the ACR scoring lexicon more strictly than experienced readers. Indeed, a recent study by Daniels et al [36] reported the worst intra-reader agreement in the most experienced radiologists.

Chung et al. [24] investigated the inter-reader agreement between three experienced and three inexperienced radiologists. They reported a moderate inter-reader agreement amongst experienced radiologists (κ = 0.47, 95% CI: 0.41–0.54) and inexperienced radiologists (κ = 0.53, 95% CI: 0.47–0.58), and between them (κ = 0.47, 95% CI: 0.44–0.49). More recently, Alyami et al. [37] conducted a similar study between two radiologists on a small sample of 39 patients and reported fair to substantial agreements (κ = 0.21–0.78) using an arbitrary scoring system of five characteristics (composition, margin, shape, calcification, and vasculitis).

A recent study by de Carlos et al. investigated interobserver variability in thyroid ultrasound assessments among four endocrinologists evaluating 100 thyroid nodules [38]. They assessed ultrasound features, risk stratification using various classification systems (ATA, EU-TIRADS, K-TIRADS, and ACR-TIRADS), and the indication for fine needle aspiration (FNA). Their findings showed acceptable interobserver agreement for identifying nodules requiring cytological study but limited concordance in risk stratification and ultrasonographic characteristics, especially for margins. The Krippendorff’s alpha coefficient for margins was 0.41, indicating moderate agreement, which aligns with our finding of poor agreement in nodule margin scores between sonographers and radiologists (κ = 0.102).

Previously, only one study was found that evaluated the sonographers TI-RADS scores [39]. Fifteen sonographers were assigned to retrospectively score 100 nodules. They found fair to moderate agreement across most scores (κ = 0.21–0.52) but only slight agreement on margin (κ = 0.18, 95% CI: 0.16–0.20). The above studies underscore the inherent challenges in achieving uniformity in TI-RADS scoring, a factor that our study further elucidates by comparing different professional groups. Notably, while previous research focused on the variability among radiologists with various levels of experience and training, our study expands this understanding by including sonographers, highlighting the broader applicability of the TI-RADS system across different medical qualifications. The ROC analysis demonstrated that sonographers had a slightly higher AUC than radiologists, indicating marginally better diagnostic performance in distinguishing between benign and malignant nodules. This finding aligns with our earlier results showing higher specificity for sonographers.

Despite the good agreement noted between the sonographers and radiologists, the agreement for the nodule margin scores was poor. This finding also corresponds with the challenges noted in the literature with this score category between radiologists [23,35]. This is critical due to the importance of this category for predicting malignancy (PPV = 94%) [40]. Our study’s findings, supported by existing literature, indicate that distinguishing between ill-defined and irregular/lobulated margins can be challenging. Indeed, the ACR guidelines lack specific criteria regarding the number and extent of protrusions required to classify margins as lobulated or spiculated. Updating the ACR whitepaper [41], published in 2015, for the TI-RADS reporting lexicon of nodule characteristics to clarify any ambiguous definitions is warranted [42]. This may potentially improve the overall agreement of the TI-RADS score. Moreover, to improve the overall reproducibility within and between readers we recommend conducting practical training on the ACR TI-RADS user guide and lexicon to eliminate personal interpretation biases. This has demonstrated its effectiveness in previous studies [24,43].

Unlike most previous agreement studies, which lack histological confirmation, the current study investigated the diagnostic performance of ACR TI-RADS scores between sonographers and radiologists. While the sonographers appear to have a better diagnostic performance, this should be cautiously interpreted for two reasons. Firstly, a key limitation of our study is that it only included nodules classified as TR3 and above by radiologists, as we focused on samples with available histological Bethesda data. This means that our findings might not fully represent the diagnostic performance across the entire spectrum of thyroid nodules, particularly those in the lower-risk categories (TR1 and TR2), which are often evaluated in clinical settings. Moreover, it is essential to note that TI-RADS is primarily a system for risk-stratification for further investigation, such as fine needle aspiration (FNA), rather than a direct diagnostic tool for thyroid malignancy. This context is crucial because it frames the system’s role in guiding clinical decisions about the need for more invasive procedures. Another limitation of our study is its retrospective design and relatively small sample size, which may introduce selection bias. By including all consecutive eligible patients and strictly defining our criteria, we attempted to mitigate this risk. Future prospective studies with larger cohorts are recommended.

Our study adds to the understanding of how TI-RADS is scored by sonographers and radiologists. The use of TI-RADS scoring by sonographers may offer several advantages, such as reducing the need for radiologist intervention in routine cases, optimizing the triage of patients requiring further evaluation, and potentially shortening the time to diagnosis and treatment [44]. Moreover, accurate TI-RADS scoring by sonographers could contribute to more cost-effective healthcare delivery, as well as reducing the overall burden on radiology departments [45]. Empowering sonographers to perform TI-RADS interpretation could enhance their job satisfaction by recognizing their expertise and expanding their professional roles. This practice also improves clinical care efficiency by streamlining workflows, reducing radiologists’ workload, and allowing for more timely patient management. Additionally, advances in artificial intelligence (AI) and deep learning have the potential to impact TI-RADS scoring. Recent studies suggest that AI algorithms can enhance diagnostic accuracy and consistency in thyroid imaging by providing objective assessments and reducing interobserver variability [46]. Incorporating AI into TI-RADS evaluation could further optimize workflow efficiency and support clinicians in decision-making.

Future research should aim to include a wider range of TI-RADS categories and work on reducing the variability in interpretation to improve the system’s effectiveness in clinical decision-making. Finally, we recommend investigating the benefits and drawbacks of enabling conclusive sonographers’ TI-RADS scores in future research, which can yield valuable insights of the outcomes.

## Conclusions

Comparing ACR TI-RADS scoring between sonographers and radiologists revealed moderate agreement, highlighting some challenges in achieving inter-reader reproducibility in thyroid nodule assessment. The poor agreement in nodule margin scores emphasizes the need for clearer lexicon definitions and training. The diagnostic performance of the sonographers’ TI-RADS scores was marginally superior to that of the radiologists when evaluated against histological outcomes. Our research, centered on suspicious nodules, highlights the importance of sonographer involvement in preliminary assessments, potentially enhancing efficiency in clinical settings. Future studies should aim to encompass a broader range of TI-RADS categories and focus on minimizing scoring variability.

## Supporting information

S1 Table Distribution of the TI-RADS features based on the Bethesda subcategories. (DOCX)

S1 Dataset (XLSX)
