# Supplementary material for: Evaluating diagnostic accuracy and agreement of TI-RADS scoring in thyroid nodules: A comparative analysis between sonographers and radiologists
Source: PLoS One. 2024 Oct 11;19(10):e0312121. doi: 10.1371/journal.pone.0312121 (PMC11469501; doi:10.1371/journal.pone.0312121)
Supplement: S1 Table — (DOCX) [file pone.0312121.s001.docx]

# Supplementary material

Table S1: Distribution of the TI-RADS features based on the Bethesda subcategories.

| TI-RADS features | Description | Nodules | | |
| --- | --- | --- | --- | --- |
|  |  | **Malignant Nodule  (n = 11)** | **Benign Nodule (n = 92)** | **Indeterminate Nodule  (n = 65)** |
| Composition score (sonographer) | Composition cannot be determined 2 points | 1 (9.1%) | 0 (0.0%) | 0 (0.0%) |
|  | Cystic or almost completely cystic 0 points | 0 (0.0%) | 0 (0.0%) | 0 (0.0%) |
|  | Mixed cystic and solid 1 point | 1 (9.1%) | 19 (20.7%) | 10 (15.4%) |
|  | Solid or almost completely solid 2 points | 9 (81.8%) | 72 (78.3%) | 55 (84.6%) |
|  | Spongiform 0 points | 0 (0.0%) | 1 (1.1%) | 0 (0.0%) |
| Echogenicity score (sonographer) | Anechoic 0 points | 0 (0.0%) | 1 (1.1%) | 0 (0.0%) |
|  | Echogenicity cannot be determined 1 point | 0 (0.0%) | 4 (4.3%) | 2 (3.1%) |
|  | Hyperechoic or isoechoic 1 point | 2 (18.2%) | 47 (51.1%) | 34 (52.3%) |
|  | Hypoechoic 2 points | 3 (27.3%) | 38 (41.3%) | 25 (38.5%) |
|  | Very hypoechoic 3 points | 6 (54.5%) | 2 (2.2%) | 4 (6.2%) |
| Shape score (sonographer) | Wider-than-tall 0 points | 9 (81.8%) | 85 (94.4%) | 56 (90.3%) |
|  | Taller-than-wide 3 points | 2 (18.2%) | 5 (5.6%) | 6 (9.7%) |
| Echogenic foci score (sonographer) | Macrocalcifications 1 point | 6 (54.5%) | 22 (24.7%) | 18 (27.7%) |
|  | None or large comet-tail artifacts 0 points | 5 (45.5%) | 52 (58.4%) | 37 (56.9%) |
|  | Peripheral (rim) calcifications 2 points | 0 (0.0%) | 2 (2.2%) | 4 (6.2%) |
|  | Punctate echogenic foci 3 points | 0 (0.0%) | 13 (14.6%) | 6 (9.2%) |
| Composition score (Radiologist) | Composition cannot be determined 2 points | 0 (0.0%) | 2 (2.2%) | 0 (0.0%) |
|  | Cystic or almost completely cystic 0 points | 0 (0.0%) | 0 (0.0%) | 0 (0.0%) |
|  | Mixed cystic and solid 1 point | 1 (9.1%) | 12 (13.0%) | 10 (15.4%) |
|  | Solid or almost completely solid 2 points | 10 (90.9%) | 78 (84.8%) | 55 (84.6%) |
|  | Spongiform 0 points | 0 (0.0%) | 0 (0.0%) | 0 (0.0%) |
| Echogenicity score (Radiologist) | Anechoic 0 points | 0 (0.0%) | 0 (0.0%) | 0 (0.0%) |
|  | Echogenicity cannot be determined 1 point | 0 (0.0%) | 0 (0.0%) | 0 (0.0%) |
|  | Hyperechoic or isoechoic 1 point | 1 (9.1%) | 48 (55.2%) | 32 (49.2%) |
|  | Hypoechoic 2 points | 7 (63.6%) | 37 (42.5%) | 28 (43.1%) |
|  | Very hypoechoic 3 points | 3 (27.3%) | 2 (2.3%) | 5 (7.7%) |
| Shape score (Radiologist) | Wider-than-tall 0 points | 10 (90.9%) | 86 (93.5%) | 60 (93.8%) |
|  | Taller-than-wide 3 points | 1 (9.1%) | 6 (6.5%) | 4 (6.3%) |
| Margin score (Radiologist) | Smooth 0 points | 5 (45.5%) | 62 (71.3%) | 39 (67.2%) |
|  | Margin cannot be determined 0 points | 0 (0.0%) | 1 (1.1%) | 1 (1.7%) |
|  | Lobulated or irregular 2 points | 5 (45.5%) | 5 (5.7%) | 4 (6.9%) |
|  | Ill-defined 0 points | 1 (9.1%) | 18 (20.7%) | 14 (24.1%) |
|  | Extra-thyroidal extension 3 points | 0 (0.0%) | 1 (1.1%) | 0 (0.0%) |
| Echogenic foci score (Radiologist) | Macrocalcifications 1 point | 3 (27.3%) | 18 (19.6%) | 18 (27.7%) |
|  | None or large comet-tail artifacts 0 points | 5 (45.5%) | 42 (45.7%) | 30 (46.2%) |
|  | Peripheral (rim) calcifications 2 points | 0 (0.0%) | 6 (6.5%) | 6 (9.2%) |
|  | Punctate echogenic foci 3 points | 3 (27.3%) | 26 (28.3%) | 11 (16.9%) |
